# Age-disparity, sexual connectedness and HIV infection in disadvantaged communities around Cape Town, South Africa: a study protocol

**DOI:** 10.1186/1471-2458-11-616

**Published:** 2011-08-02

**Authors:** Wim Delva, Roxanne Beauclair, Alex Welte, Stijn Vansteelandt, Niel Hens, Marc Aerts, Elizabeth du Toit, Nulda Beyers, Marleen Temmerman

**Affiliations:** 1 South African Centre for Epidemiological Modelling & Analysis, Stellenbosch University, 19 Jonkershoek Road, Stellenbosch 7600, South Africa; 2 International Centre for Reproductive Health, Ghent University, De Pintelaan 185, 9000 Gent, Belgium; 3 Department of Applied Mathematics and Computer Science, Ghent University, Krijgslaan 281, S9, 9000 Gent, Belgium; 4 Interuniversity Institute for Biostatistics and statistical Bioinformatics (I-BIOSTAT), Hasselt University, Agoralaan - building D, 3590 Diepenbeek, Belgium; 5 Centre for Health Economics Research and Modeling Infectious Diseases (CHERMID), Centre for the Evaluation of Vaccination, Vaccine & Infectious Disease Institute, University of Antwerp, Campus Drie Eiken CDE, Universiteitsplein 1, 2610 Antwerp, Belgium; 6 Desmond Tutu TB Centre, Department of Paediatrics and Child Health, Faculty of Health Sciences, Stellenbosch University, Francie Van Zyl Road, Cape Town 7507, South Africa

## Abstract

**Background:**

Crucial connections between sexual network structure and the distribution of HIV remain inadequately understood, especially in regard to the role of concurrency and age disparity in relationships, and how these network characteristics correlate with each other and other risk factors. Social desirability bias and inaccurate recall are obstacles to obtaining valid, detailed information about sexual behaviour and relationship histories. Therefore, this study aims to use novel research methods in order to determine whether HIV status is associated with age-disparity and sexual connectedness as well as establish the primary behavioural and socio-demographic predictors of the egocentric and community sexual network structures.

**Method/Design:**

We will conduct a cross-sectional survey that uses a questionnaire exploring one-year sexual histories, with a focus on timing and age disparity of relationships, as well as other risk factors such as unprotected intercourse and the use of alcohol and recreational drugs. The questionnaire will be administered in a safe and confidential mobile interview space, using audio computer-assisted self-interview (ACASI) technology on touch screen computers. The ACASI features a choice of languages and visual feedback of temporal information. The survey will be administered in three peri-urban disadvantaged communities in the greater Cape Town area with a high burden of HIV. The study communities participated in a previous TB/HIV study, from which HIV test results will be anonymously linked to the survey dataset. Statistical analyses of the data will include descriptive statistics, linear mixed-effects models for the inter- and intra-subject variability in the age difference between sexual partners, survival analysis for correlated event times to model concurrency patterns, and logistic regression for association of HIV status with age disparity and sexual connectedness.

**Discussion:**

This study design is intended to facilitate more accurate recall of sensitive sexual history data and has the potential to provide substantial insights into the relationship between key sexual network attributes and additional risk factors for HIV infection. This will help to inform the design of context-specific HIV prevention programmes.

## Background

In South Africa, 16.9% of the 15 to 49 years old population is infected with HIV [1]. The high prevalence of HIV in South Africa can be at least partially attributed to widespread poverty and labour migration from rural areas to urban centres that has subsequently upset family structures and disrupted psychosocial support systems [2-4]. Furthermore, continued segregation of communities along racial lines is a remnant of the old Apartheid political system and it has been suggested that this has mostly confined the epidemic to black and to a lesser extent coloured communities (the term used to describe the racially mixed descendents of Europeans, indigenous populations and slaves from South and East Asia) [5]. While the overall HIV prevalence is similar among men and women, more than 1 in 5 women (21.1%) between 20 and 24 years old is HIV-infected compared to only 1 in 20 (5.1%) men in the same age group [6]. Besides physiological factors that may increase young women's susceptibility to HIV acquisition, their frequent engagement in age-disparate relationships is assumed to impose additional risk of HIV infection. Indeed, recent data suggests that women in sexual relationships with men five or more years older are more likely to be HIV infected [1,7,8].

For young women in South Africa, an older partner is more likely to expose them to the virus, since the HIV prevalence in men increases with age, peaking in their early thirties [6]. Moreover, older men who have sexual relationships with younger women facilitate HIV transmission because they are less likely to use condoms consistently than men from the same age cohorts who do not have age disparate relationships [9,10]. Lastly, older male partners of young women frequently partake in multiple, concurrent relationships with casual partners while maintaining 'long-term' partnerships [11].

In concert with age-disparate relationships, concurrent relationships - overlapping sexual partnerships where sexual intercourse with one partner occurs between 2 acts of intercourse with another partner - have been purported by several authors to facilitate the spread of HIV [12-18]. Contrary to this assertion, are other studies that question the evidence for concurrency as a key driver of the HIV epidemics in Southern Africa [19-21]. The protective effect that polygamy has against acquiring HIV serves as counterevidence for a positive concurrency-HIV correlation [22]. The level of connectedness of individuals to the broader sexual network, rather than the number of overlapping relationships, may be a more important determinant of the spread of HIV in communities. Having concurrent relationships, changing partners frequently, and choosing partners from a wide range of age groups and geographical locations all increase one's sexual connectedness. The degree to which these behaviours are correlated and the strength of association between these behaviours and one's HIV status remains incompletely understood.

To this end, our study will be located in three disadvantaged communities in Cape Town, South Africa. The primary objective is to determine whether HIV status is associated with age-disparity and sexual connectedness. The secondary objectives of the study are to ascertain behavioural and socio-demographic predictors of the egocentric and community sexual network structures. To accomplish these objectives we will implement novel methods that will address many of the typical challenges of obtaining unbiased sexual behaviour information.

## Methods/Design

### Study Design

We will undertake a cross-sectional, sexual behaviour survey. This study is registered with and approved by the Stellenbosch University Health Research Ethics Committee (N11/03/093) and it is a subsidiary study to the Zambia South Africa TB and AIDS Reduction study (ZAMSTAR) (ISRCTN36729271), a community randomised trial aimed at reducing the prevalence of tuberculosis in communities with a high burden of TB and HIV, by novel public health interventions.

### Study Setting

The study outlined here will take place in three peri-urban, disadvantaged communities in the greater Cape Town area with a high burden of HIV. These communities represent one predominantly black community and two racially diverse communities. They were chosen in order to gather a representative sample of the different races most affected by HIV in South Africa.

### Participants

The final primary outcome measurements of the ZAMSTAR study were collected through an HIV and TB prevalence survey, conducted in 2010. In this prevalence survey, participants provided written informed consent to complete a questionnaire on basic demographic information and medical history, to provide sputum for TB culture, and to have repeated visits by the research team. Through an additional consent procedure, participants gave consent for HIV counselling and testing. Our study will randomly select 500 previous ZAMSTAR participants in each of the three communities. In the ZAMSTAR survey, some participants chose not to have HIV testing done. For this reason, 20% of our sample per study site (100/500) will consist of participants who did not consent for HIV testing. By sampling both participants who did, and who did not, consent to an HIV test, we may be able to detect selection bias.

Study participants will be included if they are between the ages of 18 and 64 at the time of the ZAMSTAR survey, able to give informed consent, and agree to complete the study questionnaire. Individuals who do not speak English, Afrikaans or isiXhosa, or who are enrolled in any other ongoing study, will be excluded.

### Measurement Instruments

A touch screen questionnaire, utilizing an audio computer-assisted self-interviewing (ACASI) application has been developed to facilitate the collection of sexual history data. The respondents will wear headphones to hear questions in their choice of English, Afrikaans, or isiXhosa, and simultaneously be able to read the question and select their answers on a 22-inch touch screen monitor. This question format was derived from a UNAIDS "Best Practice" questionnaire of sexual partnership data [23], as well as the Relationship History Calendar (RHC) and an Events History Calendar (EHC) [24]. These calendars have been previously validated and tested in adolescent and adult respondents in sub-Saharan Africa [24,25] and the United States [26]. The modified RHC used in this study will collect detailed retrospective data on sexual histories of the participants for the year preceding the survey, for a maximum of 5 'main' sexual partners and 15 'casual' sexual partners. Before starting the actual survey, participants will view a short demonstration video and will practise using ACASI and the touch screen by answering a series of example questions.

The questionnaire is set up to ask questions along a temporal trajectory. It will begin by asking the participant basic demographic information and then proceed to ask if the participant had a main sexual partner one year ago and if that relationship is still ongoing. The participant will be able to indicate, on a touch screen timeline, the periods they were in this relationship. For each relationship, we ask whether or not the participant or his/her partner used drugs or alcohol at their first intercourse. To gauge the level of spatial connectedness, we ask how long the participant usually travelled to their partner and his/her degree of proximity. For each distinct period that the respondent engaged in sexual activity with a particular partner, measured in weeks, additional questions will be asked about the frequency of intercourse and condom use. This series of questions will be asked for each main sexual partner, as well as the casual sexual partners. The onset, dissolution, and duration of each relationship will be displayed on the touch screen timeline for the participant to see, using different colours for each partner. At the end of the questionnaire, participants will be asked how many partners they have ever had. In addition to the sexual behaviour questionnaire we added three questions to assess the ease of use, perceived confidentiality and self-reported truthfulness when answering questions in this survey. Finally, we ask them to indicate their preferred medium of answering questions about sexual behaviour: touch screen computer with ACASI, researcher-administered verbal questionnaire, self-administered written questionnaire, or telephonic survey.

All questions were developed and refined after conducting cognitive interviews with six people who were representatives of the study communities. These interviews assessed the clarity, comprehensibility, and cultural sensitivity of the proposed questions, allowing us to improve the phrasing of questions, incorporate meaningful slang, and define suitable categorical answer options that maximize the precision and accuracy of responses. Furthermore, the cognitive interviews allowed us to gauge the anticipated community response to a survey utilizing ACASI. Our interlocutors responded positively to the idea of answering questions about their sexual histories in a completely anonymous way and articulated that the communities would embrace a survey that could accomplish this.

### Survey administering and data management

The senior data manager will have access to ZAMSTAR study participant information and will therefore be able to obtain names and addresses for each individual. Consent forms will be printed that include a new unique barcode - different from but linked to the barcode allocated in the ZAMSTAR study - along with names and addresses of the individuals. The barcodes from the ZAMSTAR study will not be printed on these consent forms. Therefore, none of the study staff, except the data manger, will be able to link the HIV test results obtained in the ZAMSTAR survey to our survey data.

Residents of the community, who were confirmed with names, addresses and dates of birth to be previous ZAMSTAR paricipants, will be asked if they consent to participation after reading an information sheet and listening to verbal information given by the research assistants in their home language. The research assistants will make it known that the anonymised HIV test results from the ZAMSTAR study will be accessed and used in the analysis. If the participant consents to the survey and signs the consent form, he/she will be escorted to a camper van - located in a safe part of the neighbourhood - where the survey will commence. The camper van has been refurbished to provide a private and safe office space, containing a partition, in which two participants can take the survey simultaneously at their own desk, chair and computer. Inside the van, the research assistant will scan the barcode on the consent form into the touch screen computer.

All subsequent answers to the touch screen questionnaire will be answered in private by the participant only. No one will be able to see their responses, including the research assistants who will be waiting outside of the van or in the driver's cab. They will remain close by at all times in the event that a participant requests their assistance. While two participants take the survey, two additional research assistants will be out recruiting the next two participants from the same community. The participant's name will not be asked in the questionnaire, hence the senior data manager will be the only person who can link the name from the consent form via the unique barcode. The touch screen questionnaire data will be uploaded from the laptop onto the central database designed for this study. All data will be backed up onto a second server. The consent forms will be stored in locked cabinets at the study centre.

To ensure the quality of the data collection process, the fieldwork coordinator will randomly select 5% of the completed consent forms every week and do a home visit to verify that the participant was in fact enrolled in the study and to confirm the signature on the consent form.

### HIV Status

The HIV status of participants was determined in the 2010 ZAMSTAR Tuberculosis Prevalence Survey. In that study, for those who consented to HIV testing, Abbott Determine HIV-1/2 screening tests were used, and a second, confirmatory test was conducted for those who tested positive. The data manager of this study is the only person to have access to the ZAMSTAR HIV test results and after the completion of our survey, he will link these test results to the new survey data.

### Statistical Analyses

In an initial descriptive analysis, the prevalence of HIV infection and the point prevalence of concurrent relationships and age-disparate relationships will be calculated, along with the average number of partners per year, the average frequency of sexual intercourse, the frequency of condom use for each of the reported relationships, condom use at last sexual intercourse with each of the reported partners, the average age difference between individuals and their partners and the variance of these age differences in the study population. Since many individuals are likely to report on more than one relationship, linear mixed-effects models will be used to analyze the inter- and intra-subject variability in the age difference between sexual partners, and covariates associated with large age differences. To take into account the fact that multiple sexual partnerships may co-exist, and the timing of partnership initiation and dissolution are correlated, or dependent on each other, survival analysis for correlated event times will be conducted to model concurrency patterns, partnership durations and rates of partnership initiation and termination. This type of survival analysis also accommodates right-censored observations where current partnership durations are only known up to the time of the survey. Marginal survival models for correlated event times will be used to enable comparisons between genders or other covariate groupings.

Next, logistic regression models for clustered data (due to respondents reporting on the characteristics of multiple partners, constituting repeated measures) will be fit to the data to determine whether HIV status is associated with age-disparity and sexual connectedness. Age-disparity and sexual connectedness will be operationalised using the following individual and community characteristics: (a) the mean difference between the age of an individual and the age of his/her partners; (b) the variability in age difference between an individual and his/her partners; (c) being engaged in a concurrent relationship; (d) having ever engaged in concurrent relationships; (e) the number of past and present concurrent relationships; (f) the cumulative overlapping time engaged in concurrent relationships; (g) the number of lifetime sexual partners; (h) spatial proximity to sexual partners; (i) the population mean age differences between individuals and their partners; (j) the population variability in age difference between individuals and their partners; (k) point prevalence of concurrency; (l) prevalence of having ever engaged in concurrent relationships; (m) population mean number of past and present concurrent relationships; (n) the population per capita cumulative overlapping time engaged in concurrent relationships; (o) population mean number of lifetime partners; (p) population spatial assortativeness (i.e. choosing partners from one's own community).

In a supplementary analysis, socio-demographic and behavioural predictors of egocentric and community sexual network structures will be ascertained. Potential risk factors under consideration are: race, gender, proximity to city centre, education level, socio-economic status, age, religion, and alcohol and drug use at first intercourse. Confirmed risk factors will be added to the models as confounders in the primary analyses and adjusted associations for age-disparity, concurrency and HIV status at the individual and cluster level will be calculated.

### Sample size calculations

For an alpha level of 5%, a design effect of 2.0 (effect of clustered data rather than independent random sample) and a prevalence of HIV infection of 15%, with the width of the 95% confidence interval at 6% (i.e. +/-3%), 1089 study participants with HIV test results are needed. Building a 10% margin for inconsistent and incomplete data, we will aim to administer the survey to 1200 study participants for whom HIV test results are available. Additionally, we will include 300 study participants (20% of the total sample) who did not opt for HIV testing, to investigate whether non-universal consent for HIV testing may have introduced selection bias.

## Discussion

This study will describe age-disparity and sexual connectedness in disadvantaged communities around Cape Town, South Africa. These aspects of sexual networks appear to be important for the spread of HIV, yet their complex patterns and the way in which they are driving HIV transmission are insufficiently understood. One of the fundamental challenges to understanding the role of sexual network structure in the spread of HIV is obtaining valid sexual history data. We intend to address this challenge by using a survey design that attempts to minimize social desirability bias and maximize confidentiality.

Specifically, we created a mobile office space where participants are free to answer questions away from their homes, where they often encounter many familial disruptions and potential eavesdroppers that could influence how they answer questions about their sexual behaviours. Secondly, we decided to use ACASI methods, which have successfully been used in previous sexual behaviour surveys to reduce social desirability bias. This bias is introduced when participants are intimidated by the researchers or fieldwork assistants and experience pressure to provide socially accepted answers, not necessarily in line with the truth. Studies comparing ACASI methods with face-to-face interviews, suggest that participants are more likely to report high-risk sexual behaviours while using the ACASI [27-31].

In addition to this, our study will also attempt to curb recall bias about certain sexual partners and behaviours. Events History Calendar (EHC) methods have the potential to unlock autobiographical memory better than traditional survey methods [32] because they facilitate the recollection of complex life history data [33] and they supply a constant visual cue that enhances a participant's ability to precisely recall timing of events [32,34].

This survey will expand the body of knowledge on sexual and behavioural determinants of HIV infection, with special focus on the role of age disparity and sexual connectedness. Since the population impact of an HIV prevention method does not only depend on its intrinsic efficacy, but also on the structure of the sexual network in which it is introduced, our study hopes to contribute to the development of appropriate HIV preventative strategies that take the local sexual network structure into account.

## Competing interests

The authors declare that they have no competing interests.

## Authors' contributions

WD, AW, ED, NB and MT were responsible for the conceptual design of the study. All authors participated in revisions to the study design and approved the final study design. RB and WD were involved in drafting of the manuscript, all other authors were involved in overall revision of the manuscript. All authors are involved in the implementation of the project, and have read and approved the final manuscript.

## Pre-publication history

The pre-publication history for this paper can be accessed here:

http://www.biomedcentral.com/1471-2458/11/616/prepub
